# Diagnostic digital phenotyping in schizophrenia-spectrum disorders: a systematic review

**DOI:** 10.1038/s41746-025-02194-w

**Published:** 2025-12-01

**Authors:** Ivan Vecchio, Lucas Mifsud, Sofia Castro e Almeida, Johannes Passecker

**Affiliations:** Institute of Systems Neuroscience, Medical University of Innsbruck, Innsbruck, Austria

**Keywords:** Neurological manifestations, Diagnostic markers, Cognitive neuroscience, Schizophrenia

## Abstract

Digital phenotyping offers a promising but heterogeneous approach for assessing schizophrenia-spectrum disorders (SSD). This systematic review, the first of its kind, comprehensively analyzes the diagnostic and predictive utility of digital phenotyping in SSD. Following PRISMA guidelines, we synthesized data from 142 peer-reviewed studies (2004–2024; *n* = 6294 participants). Results show a predominance of smartphone and wearable-based approaches, with only ~20% of studies combining active and passive methods. Among six symptom domains, cognitive performance yielded the largest pooled effect size (Hedges’ *g* ≈ 1.20) for differentiating individuals with SSD from controls, followed by behavior and activity (*g* ≈ 0.62). However, both domains exhibited very high heterogeneity (I² > 70%). Correlations with the PANSS scale were scarce (<5% of studies), with coefficients reaching 0.6. Relapse prediction models showed promise, with some AUC values reaching 0.8, but lacked methodological standardization. This review highlights the potential of specific digital measures while underscoring the urgent need for improved reporting, multimodal data integration, and longitudinal studies with diverse populations to enhance diagnostic and predictive power in SSD.

## Introduction

Schizophrenia-spectrum disorders (SSD) present significant diagnostic challenges. Characterized by a heterogeneous mix of positive symptoms (e.g., hallucinations, delusions), negative symptoms (e.g., social withdrawal, reduced motivation), and cognitive deficits (e.g., impaired memory, attention, executive function)^1–3^, SSD diagnosis often relies on subjective clinical assessments and patient self-reporting. Symptom severity can fluctuate considerably for individual patients, and patients vary greatly in both symptom presentation and trajectories^4,5^, making accurate and timely diagnosis difficult. The absence of well-established biological biomarkers^6,7^ further complicates the diagnostic process, leading to delays in treatment initiation and potentially poorer outcomes. Consequently, treatment selection and management in SSD present a significant ongoing challenge^8^, with treatment resistance being a major issue^9^. Tools that facilitate earlier identification of SSD, improve patient stratification, or provide predictive treatment information are highly sought after.

Digital phenotyping - the collection and analysis of behavioral data via smartphones, wearables, and other digital tools - is now integral to modern life^10^ and offers a potential solution to these diagnostic challenges. Digital phenotyping provides objective, ecologically valid insights into an individual’s daily functioning by leveraging active inputs requiring direct participant involvement (e.g., self-reports, cognitive tasks) and/or passively collected behavioral, physiological, or activity data (e.g., movement, sleep quality, social interactions) gathered automatically without active interaction.

For example, in Major Depressive Disorder, the most common mental health condition worldwide, traditional methods such as clinical interviews or self-report questionnaires often miss fluctuations in mood and behavior between appointments. Consequently, smartphones and wearables have been widely studied for their ability to provide continuous, objective monitoring of phenotypic changes over time (e.g., phone usage time, physical activity, sleep patterns, social interactions)^11–13^.

Despite the potential of digital phenotyping and these promising results in other areas of mental health^14–16^, its diagnostic utility in SSD remains unclear due to heterogeneous methods, inconsistent reporting, and a lack of comprehensive synthesis of existing research. For example, smartphone-based data, including GPS tracking and communication logs, have provided insights into social engagement levels, helping to identify patterns of social withdrawal^17^.

Ecological momentary assessments (EMA) have also been used to capture real-time emotional states and belief convictions, offering valuable data on daily fluctuations^18^. Furthermore, cognitive tests administered via smartphones and wearables have effectively measured attention and memory, with app-switching behavior and response times serving as indicators of cognitive performance^19^, and have shown promise in other mental health disorders^20,21^. However, the implementation and analysis of these technologies vary widely.

This systematic review provides, for the first time, a collective presentation of digital phenotyping approaches in individuals with SSD to facilitate comparisons across technologies, inform clinicians, and identify reporting and usage gaps to improve future studies. This analysis aims to synthesize two decades of research on digital phenotyping in SSD, highlighting key publications, challenges, and future directions to inform both research and clinical practice.

## Results

### Search results

A total of 382 studies were initially identified across four scientific databases: PubMed (*n* = 168), PsycINFO (*n* = 105), IEEE Xplore (*n* = 87), and ACM Digital Library (*n* = 9). An additional 13 studies were manually added through citation tracking of identified studies. After removing 68 duplicate records, 314 unique studies remained for screening. The first screening, based on titles and abstracts, resulted in the exclusion of 146 studies that did not fulfill the inclusion criteria, focused on non-diagnostic interventions (e.g., therapy), lacked necessary data for analysis, or used analog rather than digital methods. The remaining 168 studies underwent full-text screening, during which an additional 26 studies were excluded for not reporting data, not having a digital phenotyping focus, or because the full text was unavailable (*n* = 2). This resulted in a final selection of 142 studies that met the inclusion criteria. The full study selection process is illustrated in Supplementary Fig. 1.

### General characteristics of included studies

**Fig. 1:**
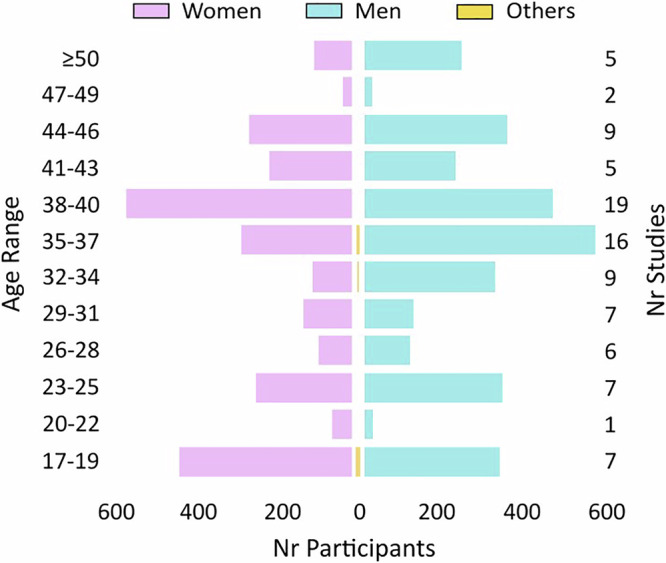
Age and sex distribution of individuals with SSD across studies. Age distribution, grouped by sex (purple = women, turquoise = men) and reported individuals identifying differently and without a report on sex (yellow = other), shows peaks in late adolescence (ages 17–19) and late thirties (ages 35–40). Inclusion was relatively balanced across sexes. Participants in their twenties and over 50 were underrepresented. The number of studies contributing to each age group is shown on the right.

Publication frequency increased significantly over time, with 91.5% (*n* = 130) of the included studies published in the last decade (2015-2024) versus only 8.5% (*n* = 12) between 2004 and 2014. Of the 142 studies, 105 were primary studies collecting data from SSD patients, and the remaining 37 were secondary or computational modeling studies. After removing duplicate datasets, the total sample size of individuals with SSD across all included studies was 6294. This total excludes participants identified through social media platforms, whose data are provided separately in Supplementary Data 1. The most frequently reported diagnoses were schizophrenia (80.9%, *n* = 115) and schizoaffective disorder (41.5%, *n* = 59). Treatment setting was stated in 48.6% (*n* = 69) of the studies. Among these, outpatients alone were the most commonly studied group (66.6%, *n* = 46), followed by studies examining both inpatients and outpatients (21.7%, *n* = 15). A smaller number of studies (11.6%, *n* = 8) focused exclusively on inpatients. Reporting of demographic and clinical characteristics varied considerably. Age and sex were the most consistently reported variables, while any information on medication was included only in half (50.5%, *n* = 53) of the primary data studies (*n* = 105). Ethnicity was reported in 29.6% (*n* = 42) of the publications; of these, 47.6% (*n* = 20) included primarily Caucasian participants, 45.2% (*n* = 19) African-American, 4.8% (*n* = 2) Asian, and 2.4% (*n* = 1) did not clarify the majority ethnicity. 50% (*n* = 71) of the studies reported education-related data. Across these studies, the mean years of education was 12.5 years (*n* = 38 datasets). The age distribution of study participants (Fig. 1) showed two peaks, one around late adolescence and another one in the late thirties (ages 35–40). Participants in their twenties and those over 50 were underrepresented. Significantly more men (53.3%, *n* = 3058) than women (46.2%, *n* = 2651) were included in the respective studies (X-squared²=29.015, df=1, *p*-value = <0.001). Nine individuals (0.16%) did not disclose their sex, and 16 reported a non-binary classification. Among the studies that reported age and standard deviation data, we observed a broad distribution of reported mean ages. The weighted mean participant age was 25.2 (*n* = 92 datasets). Additionally, studies that reported the duration of illness (17.6% of individual datasets, *n* = 25) reported a mean of 9.6 years (*n* = 24). This suggests that many individuals included in the studies had long-term illness trajectories, in line with the higher number of outpatients.

**Fig. 2:**
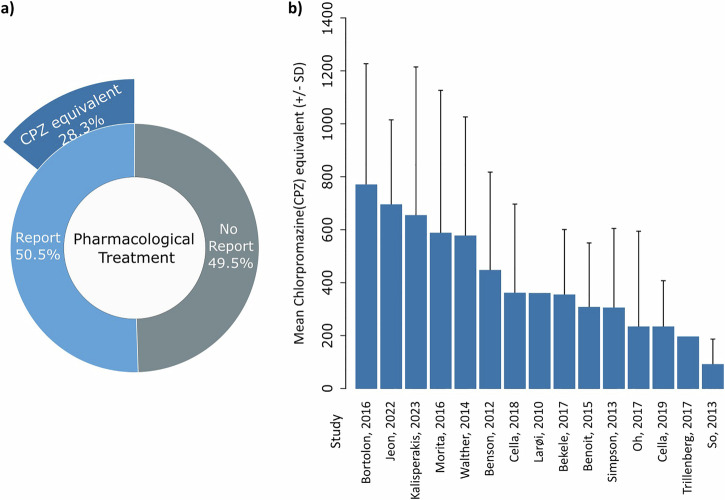
Limited treatment reporting across studies. **a** Only 50.5% of studies with primary participant data reported pharmacological treatment regimens. Of those reporting treatment, 28.3% provided chlorpromazine (CPZ) equivalent dosage information. **b** Mean reported CPZ-equivalent dosages differed widely across studies, ranging from approximately 95 mg/day to 770 mg/day (data as mean ± SD). Note. Larøi et al. and Trillenberg et al. did not provide SD.

Among the studies reporting information on pharmacological treatment, 28.3% provided chlorpromazine (CPZ) equivalent dosages (Fig. 2a). The reported CPZ-equivalent dosages varied widely, with mean daily doses ranging from approximately 95 mg to over 770 mg. The mean CPZ equivalent dosage across studies was 313.45 mg/day (*n* = 13). Standard deviations were notably large in most studies, indicating considerable within-study variability in prescribed doses (Fig. 2b).

### Digital phenotyping approaches and modalities

**Fig. 3:**
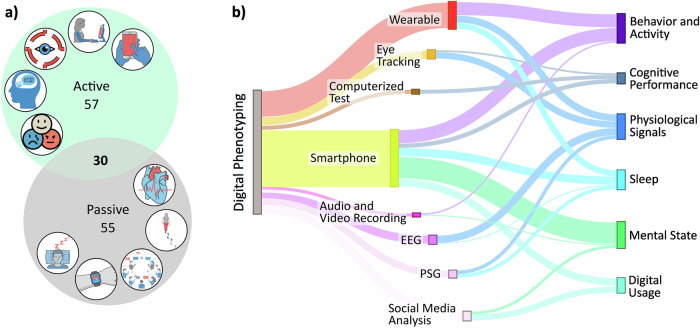
Reported phenotyping strategies for SSD symptom assessment. **a** Distribution of digital phenotyping approaches across 142 reviewed studies. Studies were categorized as using active assessment (e.g., self-reported questionnaires via app), passive assessment (e.g., sensor-based data collection), or a combination of both. **b** Breakdown of assessment modalities within active and passive approaches, based on technology and symptom-related measures.

Studies employed a combination of active and passive digital phenotyping approaches to assess SSD symptoms. Active assessments, requiring user input (e.g., self-reported questionnaires, cognitive tasks), were utilized in 57 studies, while passive assessments, relying on sensor-based data collection, were applied in 55 studies. Additionally, 30 studies combined both methods, leveraging their complementary advantages (Fig. 3a). Studies incorporating both active and passive approaches primarily relied on smartphones that gathered both active measurements and passive sensor data (53.5%). Smartphones were the most frequently used tool across digital phenotyping modalities, particularly in active assessments. Here, smartphone-based cognitive tasks and surveys (61.4%) were the predominant modality. They were primarily employed to measure mental state and cognitive performance, relying on self-reported data, cognitive tasks, and app-based assessments. In passive assessments, smartphones were used to monitor behavior, activity patterns, digital usage, and sleep through built-in sensors (e.g., accelerometers, light sensors, microphones, GPS). Wearables, on the other hand, were the predominant tool (45.5%) for passive assessments, offering continuous, real-time monitoring of physiological and behavioral measures. They captured movement and activity levels through accelerometers and gyroscopes, while also playing a significant role in sleep assessment, measuring heart rate variability, body movement, and sleep-wake cycles. Additionally, physiological signals (e.g., heart rate, skin conductance, temperature) were frequently measured to assess autonomic nervous system activity, linked to stress regulation and emotional states in SSD. EEG provided objective insights into cognitive function, brain function, and sleep architecture, while PSG offered in-depth measurements of sleep disturbances. Social media analysis focused on linguistic markers, sentiment analysis, posting patterns, and communication behaviors, helping to identify thought disturbances, social withdrawal, and mood fluctuations (Fig. 3b).

### Digital phenotyping measures and clinical correlations

**Fig. 4:**
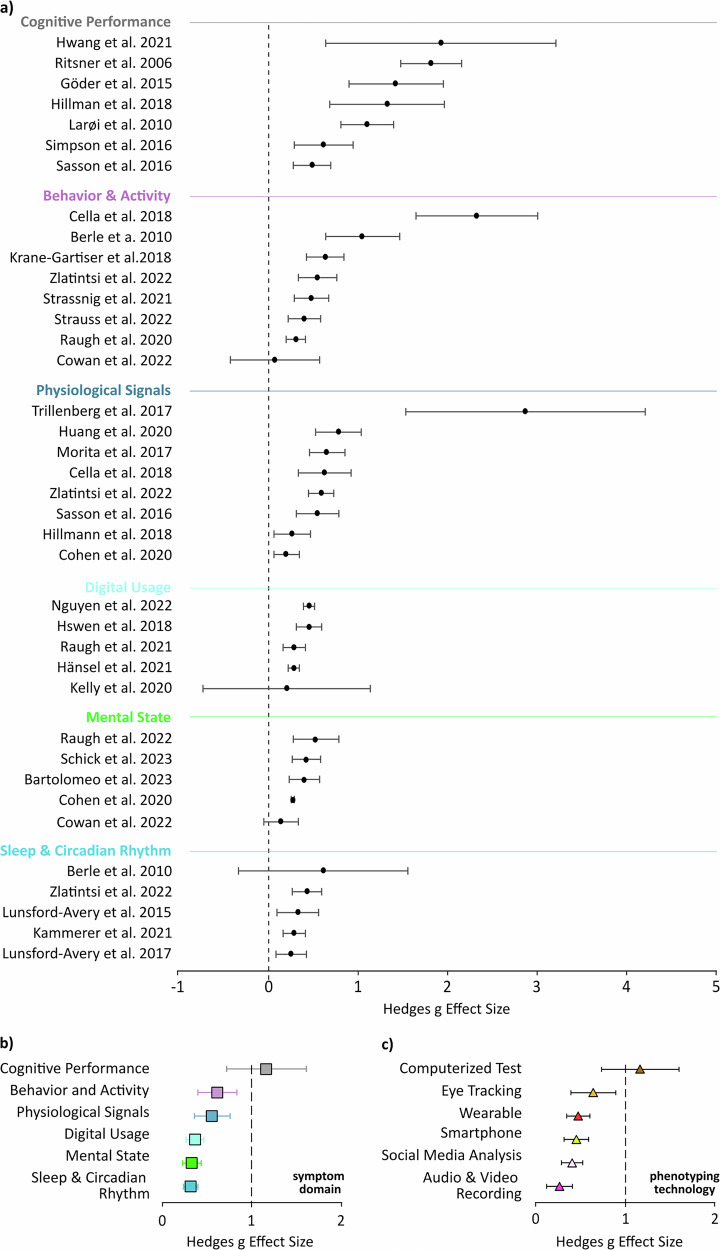
Effect size comparison of digital phenotyping measures between individuals with Schizophrenia-Spectrum Disorder and healthy controls. The figure displays: **a** the aggregated mean effect size for each study, grouped by symptom domain (colors); **b** the weighted average effect size for each of the six symptom-related domains; and **c** the weighted average effect size for each digital phenotyping technology. (Data as Hedges’ g ± 95% CI; individual measures are listed in Supplementary Data 3).

Next, we aimed to determine which of the six symptom-related domains - cognitive performance, physiological signals, behavior and activity, sleep and circadian rhythm, mental state, and digital usage - presented the most reliable and strongest differentiators between HC and individuals with SSD. Figure 4 summarizes the effect size analyses at different levels of aggregation. Panel 4a provides an overview of the aggregated mean effect size for each individual study, categorized by symptom domain. These study-level means are then summarized at the domain level in panel b and aggregated by phenotyping technology in panel c. In contrast, Supplementary Fig. 3 visualizes only the individual measurements with a large Hedges’ g effect size (*g* ≥ 0.8), with further aggregations per symptom domain (Supplementary Fig. 3b) and phenotyping device (Supplementary Fig. 3c). The complete list of all 336 individual measurements and their calculated effect sizes is provided in Supplementary Data 3.

The cognitive performance domain yielded the strongest, yet variable, pooled effect size (Hedges’ *g* = 1.171, 95% CI: 0.7317-1.6103, *n* = 7 studies; Fig. 4b). Within this domain, measures of emotion identification and attention indices exhibited some of the largest individual Hedges’ g values. Notably, two studies with high effect sizes, Hwang et al.^22^ (52 SSD, 50 HC) and Ritsner et al.^23^ (55 SSD, 63 HC) utilized composite indices that combined multiple cognitive features. The behavior and activity domain showed more moderate but robust effects, with a pooled effect size of *g* = 0.6200 (95% CI: 0.4021–0.8379, *n* = 8; Fig. 4b). For example, Cella et al. (30 SSD, 25 HC)^24^ used a wristband to measure accelerometer-based movement and heart rate variability over six days, while Berle et al. (23 SSD, 23 HC)^25^ used actigraphy over two weeks to monitor motor activity patterns like inter-daily stability and intra-daily variability. Physiological signals resulted in a pooled effect size of *g* = 0.5671 (95% CI: 0.3729–0.7613, *n* = 8; Fig. 4b). Eye-tracking metrics, such as saccade velocity and fixation patterns, showed particularly significant differences between groups (Supplementary Fig. 3a). For instance, Trillenberg et al.^26^ (21 SSD, 24 HC) reported strong effects in a foveo-fugal ramp task by systematically varying task parameters to isolate specific sensorimotor and cognitive and contributions to deficits in eye movements. The remaining domains showed weaker pooled effect sizes. These included digital usage (*g* = 0.3750, 95% CI: 0.2734–0.4765, *n* = 5), mental state (*g* = 0.3424, 95% CI: 0.2366–0.4482, *n* = 5), and sleep and circadian rhythm (*g* = 0.3298, 95% CI: 0.2497–0.4100, *n* = 5; Fig. 4b).

As expected, computerized tests, which primarily assess cognitive function, outperformed all other technology categories (Fig. 4c) but also exhibited high variability; (*g* = 1.17, 95% CI: 0.73–1.61 *n* = 7). Eye-tracking, which captures physiological responses often linked to cognitive processes, followed with a pooled effect size of *g* = 0.635 (95% CI: 0.376–0.893 *n* = 6). Wearables, Smartphones and Social Media analysis had effect sizes of around 0.4 (*g* = 0.464, *n* = 8, g = 0.427, *n* = 10, *g* = 0.397, *n* = 4, respectively), while Audio & Video recordings only presented an effect size of *g* = 0.256 (95% CI: 0.108–0.403 *n* = 2; Fig. 4c).

Heterogeneity analysis revealed substantial variability in effect sizes across studies within most symptom domains (Supplementary Table 1). The highest overall heterogeneity was observed for cognitive performance (I² =93%), followed by physiological signals (I² =89.5%) and the behavior and activity domain (I² = 70.08%). It was moderate for sleep and circadian rhythm (I² = 41.1%) and was lower for mental state (I² = 51%) and digital use (I² = 32.73%). The high heterogeneity even within cognitive subgroups like emotion processing (I² > 90%) likely reflects significant methodological differences in the tasks and measurements used across studies.

### Comparison between PANSS scores and digital phenotyping

**Fig. 5:**
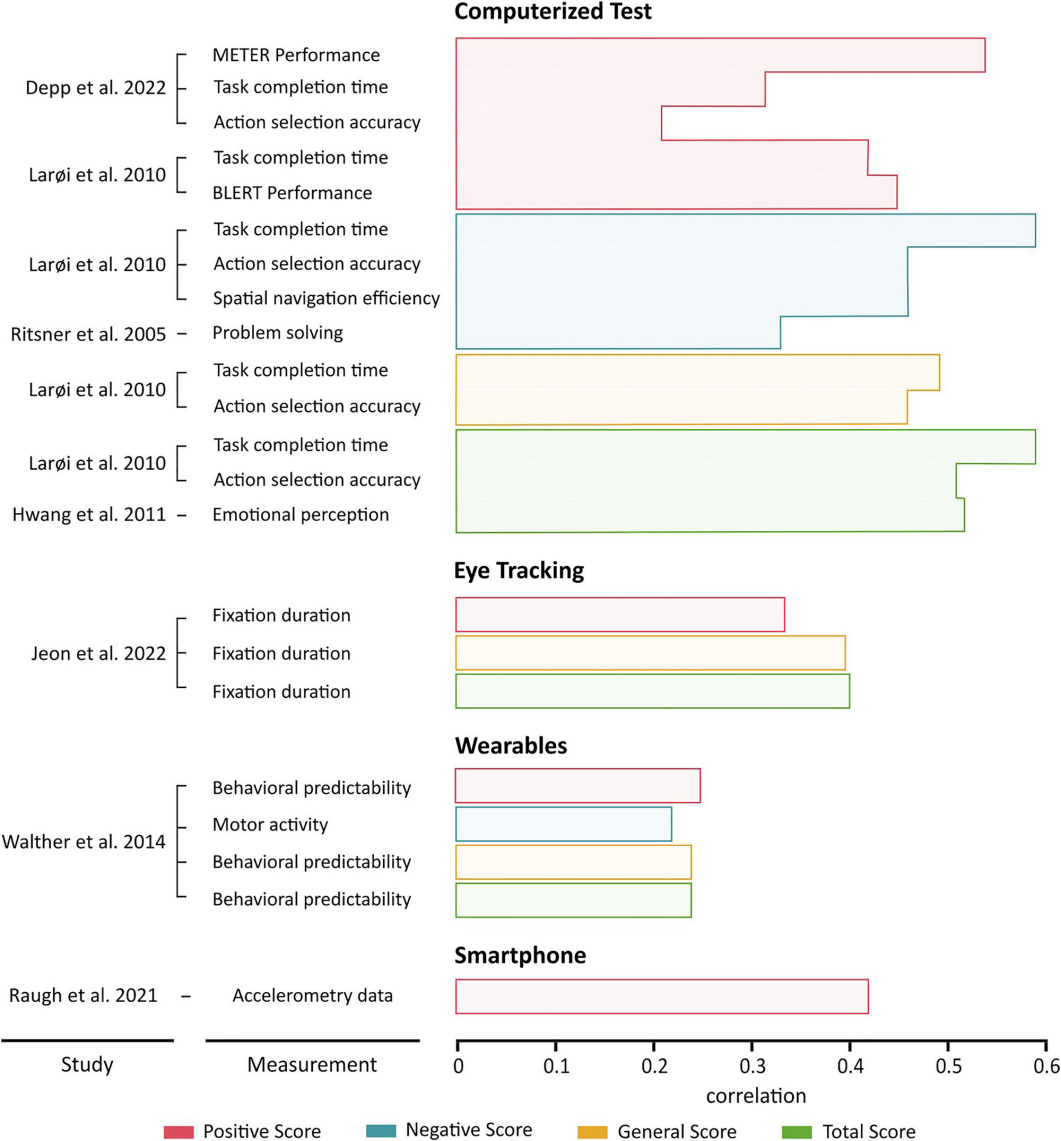
Comparison of digital diagnostic metrics against traditional PANSS subscales (Positive and Negative Syndrome Scale) at baseline. Correlations between digital diagnostic measurements and PANSS composite scores, used to categorize individuals with SSD. PANSS scores are divided into Positive (red), Negative (blue), General (orange), and Total scores (green). Significant measurements include computerized tests, eye tracking, smartphone and wearable data. Computerized tests generally showed the highest correlations, particularly for Positive and Total scores, while eye-tracking and wearable-based measurements also demonstrated meaningful, yet lower, correlations.

The PANSS is the most commonly used assessment tool for scoring the severity of SSD symptoms. Therefore, we aimed to determine whether digital phenotyping measures could be reliably correlated with PANSS scores. Only 7 of 35 studies (20%; Fig. 5) that reported PANSS scores for their participants (and 4.9% of all 142 included studies) presented a statistical correlation between their digital phenotyping findings and their clinical scale measures. Notably, these comparisons were exclusively performed at baseline, typically within 24 hours of initiating the digital data collection. The majority of these studies (five out of seven) were cross-sectional, involving short-term assessments. While two studies, Raugh et al.^27^ and Depp et al.^28^ employed longitudinal designs, they too only reported PANSS assessments at baseline. Consequently, no included study compared symptom fluctuations over time between digital phenotyping and traditional PANSS ratings.

Across the PANSS subscales (Positive, Negative, General, and Total), computerized test measurements exhibited the strongest and most consistent associations with SSD symptoms (Fig. 5). For example, measures like task completion time in a simulated shopping task- an indicator of processing speed and executive function -showed some of the highest correlations. Among the studies showing the strongest correlations, the work by Larøi et al. (2010)^29^ is notable for its ecological validity. Its use of a realistic, everyday activity provides valuable insights into how cognitive deficits affect real-world functioning, in line with previous meta-analytic findings^30^. Similarly, Depp et al.^28^ demonstrated several strengths, including the use of a computerized EMA method for daily cognitive assessments and a direct comparison between their digital assessment and established laboratory-based tasks. Together, these two studies account for 54% (*n* = 12/22) of the significant clinical correlations identified in our analysis. Furthermore, a particularly strong correlation with the total PANSS score was identified in Hwang et al. (2021)^22^.

However, a factor limiting the interpretation of these correlations is the inconsistent and often incomplete reporting of quality assurance for the PANSS assessments themselves. Across the studies utilizing the PANSS, 63% did not report on the qualifications or training of the raters, and only one study (Ritsner et al., 2006)^23^ reported on inter-rater reliability.

### Predictive performance of digital phenotyping models in psychotic relapse detection

**Fig. 6:**
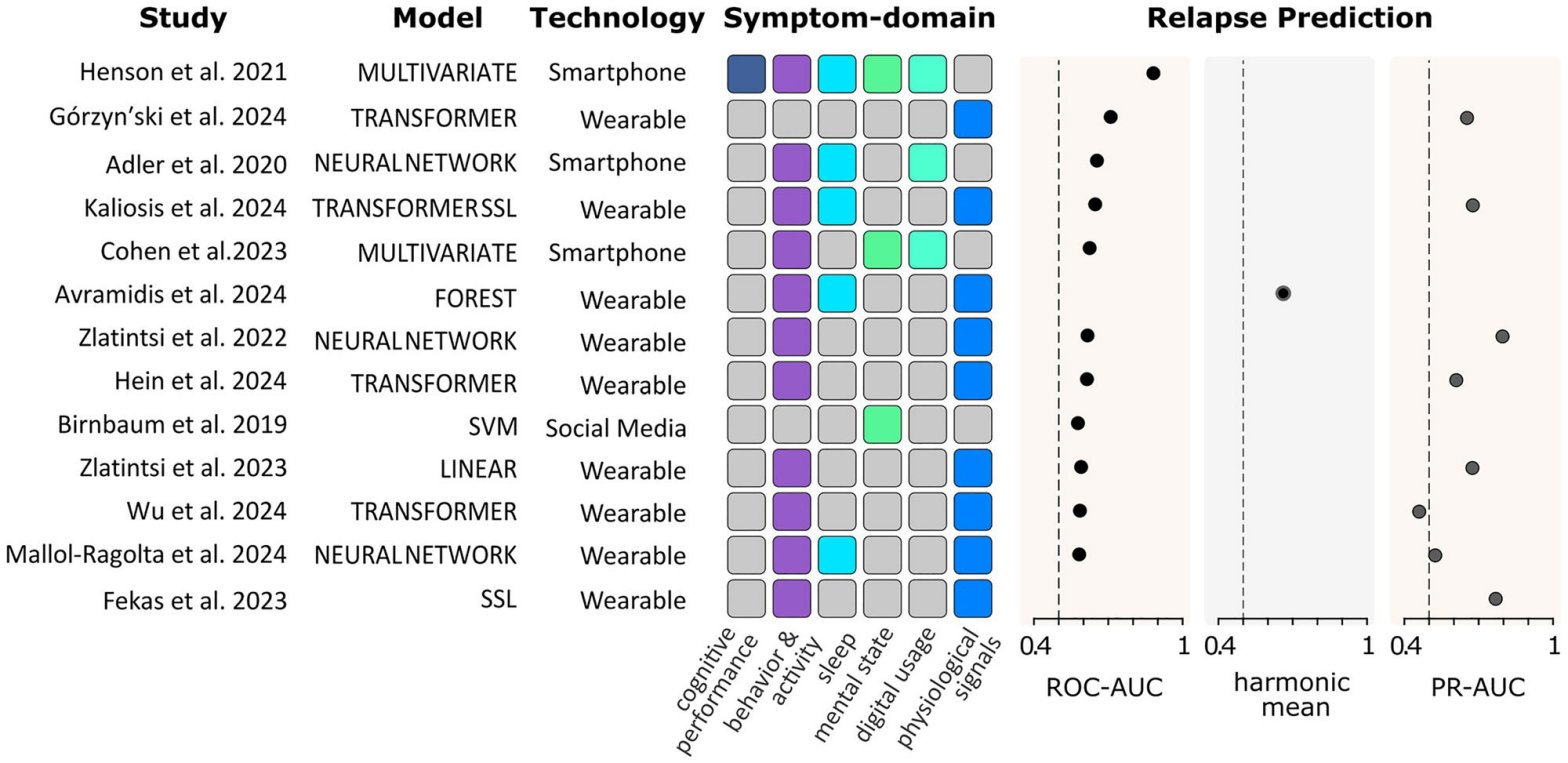
Overview of studies employing machine learning models to predict psychotic relapse using digital phenotyping data. Studies differ in the specific models used (e.g., multivariate analysis, neural networks, transformers, support vector machines) and the technology employed (e.g., smartphone-based [SP], wearable devices [W], social media analysis [SMA]). Symptom-domain features used in relapse prediction are color-coded and include physiological signals, sleep patterns, behavior and activity, digital usage, mental state, and cognitive performance. Three dot-plot panels summarize the predictive performance, with higher AUC values indicating better predictive accuracy. Extracted models include a wide range of approaches, including: Multivariate Gaussian (MULTIVARIATE), CNN autoencoders (NEURAL NETWORK), self-supervised learning combined with survival analysis (SSL), transformer-based autoencoders (TRANSFORMER), fully connected neural networks (NEURAL NETWORK), transformer-based self-supervised learning methods (TRANSFORMER SSL), multivariate analyses (MULTIVARIATE), single-layer linear autoencoders (LINEAR), isolation forest algorithms (FOREST), transformer-based models (TRANSFORMER), one-class support vector machines (SVM), gated recurrent unit and recurrent neural networks (GRU, RNN) with autoencoders (NEURAL NETWORK), and transformer-based networks with elliptical envelope for anomaly detection (TRANSFORMER). Note: for Avramidis et al. 2024 only the harmonic mean of PR- and ROC AUC is reported. Zlatintsi et al. 2022 and 2023, Górzyn′ski et al. 2024, Kaliosis et al. 2024, Hein et al. 2024, Wu et al. 2024, Mallol-Ragolta et al. 2024 analyzed overlapping datasets as part of the E-prevention challenge.

The performance of models predicting psychotic relapse was typically evaluated using the AUC, often from Receiver Operating Characteristic (ROC) or Precision-Recall (PR) curves. AUC values range from 0 to 1, quantifying the model’s ability to discriminate between relapse and non-relapse states, with higher values indicating better performance. Figure 6 summarizes the included studies, detailing the machine learning models, digital technologies (e.g., smartphones, wearables), and reported AUC scores.

These predictive models utilized various digital phenotyping tools. Passive monitoring via wearable devices was common, likely owing to their suitability for collecting longitudinal physiological and behavioral data with minimal user burden. Data frequently collected via wearables included physiological signals (such as heart rate variability and skin conductance, which are linked to stress responses and symptom fluctuations in SSD) and behavior/activity patterns. Smartphones were employed in fewer relapse prediction studies, sometimes used for active data collection or integrated within multivariate models. In general, model performance and approaches varied considerably across studies.

The first landmark study using exclusively social media data (Facebook linguistic and posting-behavior analysis), by Birnbaum et al. (2019)^31^, reported modest predictive accuracy (estimated ROC-AUC of 0.58, reporting specificity = 0.71, sensitivity = 0.38, PPV = 0.66), despite including the highest number of relapse events of any study. In contrast, Górzyński et al. (2024)^32^ achieved stronger performance (ROC-AUC = 0.71, PR-AUC = 0.62) using only physiological heart-rate metrics from a wearable device.

Generally, higher predictive accuracy was achieved in studies that integrated data from multiple symptom domains. For example, Zlatintsi et al. (2022)^33^ and Fekas et al. (2023)^34^ reported the highest PR-AUC values (0.77 and 0.745, respectively) from data collected in the E-prevention study. While the studies by Cohen et al. (2023)^35^ and Henson et al. (2021)^36^ included the largest patient cohorts (*n* = 76 and *n* = 63, respectively), they observed a relatively low number of relapse events (23 and 29) during their multi-month observational periods. Both of these studies used a multivariate approach to detect data anomalies from various smartphone-based assessments; however, they reported markedly different outcomes, with Henson et al. achieving the highest ROC-AUC of any study (0.883) and Cohen et al. (2023)^35^ reporting an ROC-AUC of 0.626. Direct comparison of these models is complicated by two key factors. First, the definition of “relapse” was inconsistent across studies, variously defined as hospitalization, an increase in a PANSS or BPRS score, the onset of suicidal ideation, or a combination of these events. Second, we observed stark differences in methodological approaches and reporting standards, which further hinder direct comparisons. To highlight this variability, we performed both a general quality assessment for all studies and a specific risk of bias analysis focused on these relapse prediction studies.

### Risk of bias evaluation

We also evaluated the quality of reporting across the included studies using six methodological criteria, rated on a 3-point scale (Supplementary Fig. 2a presents an overview; Supplementary Data 1 provides details per study). Certain aspects were generally well-reported. Balanced reporting of results scored highest, with 89.4% (*n* = 127) providing both positive and negative findings, which suggests a lower risk of selective reporting bias. Eligibility criteria and recruitment procedures were also relatively well-documented, with 73.2% (*n* = 104) and 76.8% (*n* = 109) of studies, respectively, providing full reports, although a notable portion provided only partial details. Reporting deficiencies were identified in other critical areas, participant adherence to digital technology was lacking in 21.8% (*n* = 31), and the handling of missing data was not described in 14.1% of studies (*n* = 20). Justification for sample size was largely absent, with 94.4% of studies (*n* = 134) providing no rationale. For studies performing relapse prediction, we scored their quality and risk of bias based on 25 items from the PROBAST + AI^37^ (Supplementary Fig. 2b; Supplementary Table 2). The highest scoring papers were Birnbaum et al.^31^ and Adler et al.^38^. Overall, the handling of missing data and the application of evaluation methodologies were well-described across most studies. The majority of manuscripts also provided detailed descriptions of their code or prediction model, enhancing reproducibility even when clinical data could not be shared. However, no prediction model was validated via external validation datasets, which is unsurprising given the scarcity of accessible public datasets in this field. Model fairness was not addressed in any study, a gap likely attributable to small sample sizes and the low number of relapse events. Critically, the majority of studies did not describe potential differences between the datasets used for model development and evaluation, and only two studies described any procedures for model updating. In summary, we observed significant heterogeneity in methodological approaches and reporting. More detailed and consistent adherence to reporting guidelines will be required in the future to increase the comparability and interpretability of such predictive models.

## Discussion

In recent years, digital phenotyping has gained widespread recognition as a valuable tool for monitoring behavior, health, and well-being in both clinical and non-clinical settings^11,13,14,21^. By leveraging data from smartphones, wearables, and other digital devices, it provides a valuable approach for capturing more granular and ecologically valid insights. While digital phenotyping has shown promising results across diverse mental healthcare fields - from sleep monitoring to mood tracking - its effectiveness in SSD remains unclear.

This systematic review and quantitative synthesis, encompassing 142 studies, provides the first comprehensive analysis of digital phenotyping for diagnostic and relapse prediction purposes in SSD. Our findings reveal a growing adoption of digital tools, primarily smartphones and wearables, for both active and passive data collection. The quantitative analysis showed that cognitive performance yielded the largest pooled effect size (Hedges’ g ≈ 1.20) in differentiating individuals with SSD from controls, followed by behavior and activity (*g* ≈ 0.62), although substantial heterogeneity (I² > 90% and > 70%, respectively) tempers these results. Critically, validation against standard clinical scales like PANSS remains scarce. Furthermore, while models predicting psychotic relapse show variable performance (with multimodal approaches achieving AUCs approaching 0.8), the lack of standardized relapse definitions, validation methods, as well as reporting inconsistencies hinders comparability, and such high predictive capabilities should be considered with caution. These results highlight the promise of specific digital measures but underscore the urgent need for methodological rigor and standardization across the field. Our heterogeneity analysis quantitatively confirms substantial variability across most digital phenotyping domains for SSD. A positive example is the relatively low heterogeneity observed for sleep architecture measures (I² = 5.4%), likely due to a more standardized approach and the physiologically stable nature of sleep itself. This contrast highlights that while consistency across clinical populations and medication is challenging, the choice of measurement tools, environmental setting, and methodological standardization significantly impacts the reproducibility and comparability of findings and requires significant improvement in the future. Nevertheless, the high heterogeneity observed in many domains necessitates cautious interpretation when generalizing results and strongly reinforces the need for standardized protocols in future research.

The strong performance of cognitive and physiological measures aligns with the established neurocognitive and physiological deficits characteristic of SSD^39^. The finding that digital assessments of emotion recognition^40,41^ and attention^42^ show large effect sizes is particularly noteworthy, as these domains are known to be impaired in SSD and are linked to functional outcomes.

The moderate correlations between digital measures and baseline PANSS scores, while not strong enough to suggest replacing traditional assessments, indicate that digital phenotyping can objectively capture aspects of symptom severity. This is particularly relevant given the known limitations of clinic-based assessments and the subjectivity inherent in PANSS ratings^43,44^. Our review highlights this issue, as we observed limited reporting on rater training and inter-rater reliability for the PANSS across the included studies.

Furthermore, our findings reveal a discrepancy between the large effect sizes of digital measures in differentiating individuals with SSD from healthy controls - especially for cognitive performance - and the relatively weak correlations these same measures have with PANSS scores. A likely reason for this disconnect is the limited capacity of the PANSS to adequately assess objective cognitive performance, particularly within a standard 3-factor model^45–47^. This supports concerns about the validity of using the PANSS as ground truth for validating objective cognitive and functional digital measures, as previously reported^48–51^.

The variability in relapse prediction performance highlights the complexity of this task. Relapse in SSD is likely influenced by a multitude of interacting factors, many of which are not fully captured by current digital phenotyping approaches. The success of Henson et al.^52^ in achieving a high ROC-AUC may stem from their integration of multiple data streams. Notably, their model was the only one in our comparison to include cognitive performance data that previously showed the highest differentiating factor between HC and SSD. This suggests that a holistic, multimodal approach is critical for accurate relapse prediction. However, direct comparisons are complicated by the lack of a universal definition of “relapse” across studies and differences in reporting practices. Also, in general, it is important to report time-to-event analysis and the number of false alerts per patient, as these reflect the real-world challenge of managing patient care and avoiding alert fatigue.

While our review categorized digital phenotyping technologies, it’s crucial to consider the strengths and limitations of each, specifically within the context of SSD.

Smartphones: Offer widespread accessibility and the ability to collect both active (e.g., EMA, cognitive tasks) and passive (e.g., GPS, accelerometer) data and are also in our analysis the most utilized tool (Fig. 3). However, reliance on self-report on mental state assessment can be problematic in individuals with SSD experiencing cognitive deficits or impaired insight that may contribute to the lower effect sizes presented with EMA (Fig. 4c, Supplementary Figure 3c). Furthermore, data quality can be affected by inconsistent phone usage, varying levels of digital literacy, and potential biases in who chooses to participate in smartphone-based studies. Providing more details on adherence and missing data is critical for new studies in order to better understand the strengths and limitations. In general, our analysis provides support that a stronger focus on computerized tests and behavior & activity measurements through smartphones may increase its utility, given its widespread use and acceptance.

Wearables: Provide continuous, objective monitoring of physiological and behavioral data (e.g., activity, sleep, heart rate variability). This is particularly valuable in SSD, where objective measures of negative symptoms (e.g., reduced activity) and sleep disturbances are needed. However, the accuracy of wearable sensors can vary considerably across devices and manufacturers, and proprietary algorithms used to process data can be “black boxes,” making interpretation difficult. Participant adherence to wearing devices consistently can also be a challenge.

Eye-Tracking: Offers objective measures of cognitive, visual processing and attentional processes known to be impaired in SSD. Traditionally, eye-tracking typically requires specialized equipment and controlled laboratory settings, limiting its ecological validity and scalability for widespread clinical use. However, recent developments provide new solutions, increasing the utility of smartphone-based solutions^53^.

Computerized Tests: Provide standardized and objective assessments of cognitive function. Those with high ecological validity (e.g., simulating real-world tasks) may be particularly relevant for assessing functional capacity in SSD. While this category outperformed other domains, variability is high, and more rigorous validation and reporting will be required, particularly when they are used in mobile and naturalistic settings.

Social Media Analysis: As the study by Birnbaum et al.^31^ shows, social media analysis can have the potential to provide insights into social behavior, communication patterns, and thought processes. In general, though, the category had one of the lowest effect sizes when comparing HC with SSD. The interpretation of online behavior in the context of mental illness is complex and requires careful consideration of potential biases and raises significant ethical concerns regarding privacy and informed consent^54,55^.

Many studies exhibited a moderate risk of bias, primarily due to incomplete reporting of participant characteristics (particularly ethnicity and medication details), inadequate descriptions of data collection procedures, and a lack of clear justification for statistical methods. This heterogeneity in reporting and methodological rigor introduces a potential for bias that may influence our overall findings. Specifically, the underreporting and variability of potential confounding variables (e.g., medication, comorbidities) make it difficult to isolate the specific effects of digital phenotyping measures. Our risk of bias assessment indicated that while many studies reported methodological aspects such as results, eligibility criteria, and recruitment procedures, this reporting was often incomplete (Supplementary Fig. 2a; details in Supplementary Data 1). Furthermore, the assessment highlighted critical omissions particularly relevant for evaluating digital health technologies: deficient reporting on participant adherence and the handling of missing data were prevalent across the reviewed literature.

Our risk of bias analysis for relapse prediction studies, guided by the PROBAST + AI^37^ framework, reveals critical shortcomings that temper the promising prediction accuracies reported. While aspects like the handling of missing data and model evaluation methods were generally well-described, foundational methodological elements were frequently omitted. Most notably, the broader generalizability of the proposed models is limited by the lack of external validation across studies, which is understandable given the scarcity of public datasets and the prevalence of individualized prediction models. Furthermore, the majority of studies failed to describe differences between their development and evaluation data or address potential model fairness issues, which are critical for clinical translation. While the transparency in reporting model code is a positive step, the overall heterogeneity in reporting and methodology underscores an urgent need for adherence to standardized guidelines to ensure future models are robust, comparable, and ultimately, clinically useful.

Despite these comparative limitations, this review highlights the potential of digital phenotyping to improve and complement the diagnosis and management of SSD. The findings suggest that digital phenotyping can provide objective, continuous, and ecologically valid measures that complement traditional clinical assessments for SSD patients. However, to better translate this potential into clinical practice, several key challenges must be addressed.

The inconsistent reporting and methodological heterogeneity across studies underscore the urgent need for standardization and adherence to guidelines. Establishing common data repositories, datasets^56^, standardized protocols for data collection and analysis, and clear reporting guidelines (e.g., extending CONSORT^57^ or STROBE^58^ or other guidelines^59,60^ to specifically address digital phenotyping studies) would greatly enhance the comparability and reproducibility of research findings.

Despite the known shortcomings of traditional diagnostic scales, only a limited number of studies that use them report correlations between digital phenotyping tools and established scales like the PANSS or BPRS, highlighting a significant gap between research and clinical implementation. A notable gap in the literature is the absence of direct comparisons between digital phenotyping and traditional scales for tracking symptom fluctuations over time. We found that no included study conducted such a longitudinal comparison, a limitation attributable to study designs that were either cross-sectional or restricted clinician-based ratings to a single baseline assessment. The variability in relapse prediction performance underscores the need for larger, more diverse datasets, potentially including more cognitive assessments. It also highlights the need for a consensus definition of “relapse” in the context of digital phenotyping research.

In conclusion, digital phenotyping offers a promising avenue for transforming the diagnosis, stratification, and management of SSDs, potentially addressing long-standing challenges related to subjective assessments, infrequent symptom measurements, and the lack of objective biomarkers. While our findings suggest that digital phenotyping measures can differentiate individuals with SSD from healthy controls and show potential for psychotic relapse prediction, the substantial heterogeneity in study designs, small sample sizes, data collection methods, reporting practices, and variations in relapse definition hinders definitive conclusions. Following standardized guidelines^60^ on predictive outcome measures will be critical.

To realize the full potential of digital phenotyping in SSD, and to translate research findings into tangible clinical benefits, we suggest that future research must prioritize the following:

1. Standardization: Refinement and adoption of standardized data collection and reporting protocols, including common data elements and validated outcome measures (including a consensus definition of relapse), are crucial for enhancing comparability and reproducibility across studies.
2. Diversity and Sample Size: Ensuring future studies recruit larger, more diverse, and representative populations is crucial for generalizability and equity.
3. Rigorous Methodology: Employing robust study designs, including adequate sample sizes justified a priori, comprehensive quality/bias assessments, appropriate handling of confounders and missing data, and transparent reporting is essential.
4. Multimodal Data Integration: Leveraging the complementary strengths of active and passive data streams, integrating physiological, behavioral, cognitive, and environmental data likely holds the key to improving diagnostic and predictive accuracy.
5. Longitudinal Validation: Conducting longitudinal studies that validate digital measures against established clinical scales and real-world functioning over time is critical to establish clinical utility.

By fostering interdisciplinary collaboration among researchers, clinicians, individuals with lived experience, and technology developers, and by prioritizing standardized, inclusive, and ethically sound research practices, digital phenotyping has the potential to advance the assessment and management of SSD. In clinical settings, these tools could enhance early detection, support personalized treatment, improve relapse prediction, reduce assessment burden, and ultimately, improve outcomes and quality of life for individuals with SSD.

## Methods

### Overview

This systematic review was conducted following the PRISMA (Preferred Reporting Items for Systematic Reviews and Meta-Analyses) guidelines^61^ to ensure transparency and reproducibility (see Supplementary Information).

### Search terms and strategy

Our search strategy targeted four specialized databases to reflect the interdisciplinary nature of digital phenotyping. We selected PubMed as the primary source for medical and life science research and PsycINFO for its comprehensive coverage of the psychology-related field. To incorporate the crucial technological and engineering aspects, the search also included the ACM Digital Library and IEEE Xplore, which are standard repositories in computer science. We included terms such as schizophrenia, psychosis, digital phenotyping, smartphone, and symptom monitoring (see Supplementary information for exact search terms). We used a combination of standardized keywords (e.g., MeSH terms in PubMed) and free-text keywords, along with Boolean operators (AND, OR, NOT), to maximize search results. The literature search was completed on November 28, 2024, and was restricted to articles published in English or German between 2004 and 2024 in line with the emergence of smartphones in the mid-2000s.

### Inclusion criteria, screening and selection of studies

Studies were included if they met all the following criteria: (1) included measurements of individuals with SSD; (2) were published between 2004 and 2024; (3) were written in English or German; (4) were peer-reviewed; (5) had accessible full text; and (6) utilized digitally collected data. Studies underwent title and abstract screening for relevance. Subsequently, full-text screening was performed. The initial screening and data extraction were conducted by the first author. For validation, at least one co-author independently assessed each manuscript for inclusion. Any discrepancies or uncertainties regarding study selection or data extraction were resolved through discussion and a majority decision among all co-authors. This dual-reviewer process was implemented to ensure consistency and minimize subjective bias. A PRISMA flow diagram details the complete selection process (Supplementary Fig. 1).

### Data extraction and synthesis

For each included study, relevant information was extracted from the full text and systematically recorded (details in Supplementary Data 1). Data extraction focused on primary study features, including study design, sample demographics, clinical and diagnostic variables, digital phenotyping methods, and statistical/methodological aspects. To summarize key demographic and clinical characteristics across studies, pooled mean values were calculated using the inverse variance-weighted method. This method weights each study’s mean by its precision, utilizing the reported mean, sample size (n), and standard deviation (SD). For any specific characteristic being pooled, studies that did not report the standard deviation were excluded from that particular analysis.

The extracted data were categorized into key symptom-related domains and methodological subgroups to facilitate comparison. To prevent the inclusion of duplicate datasets, we systematically compared author information and key sample characteristics (e.g., sample size, demographics) across all studies. In cases where both a preliminary report and a final study from the same research group were identified, only the final, more complete dataset was retained for analysis to avoid overlapping study populations. Despite these precautions, we cannot fully exclude the possibility that some individuals may have participated in more than one included study. An exception is the inclusion of 8 computational studies that used the same underlying patient dataset for relapse prediction; here, the data was counted only once for any descriptive analysis (e.g. demographics).

Due to the complexity of SSD, which affects multiple cognitive, behavioral, and physiological domains, we purposefully included a wide range of digital phenotyping and measurement strategies to cover these aspects as comprehensively as possible. Consequently, we categorized all studies based on both the utilized diagnostic technology and the tested symptom-related domains.

GraphPad Prism was used for statistical analysis and plots, including confidence intervals and effect size comparisons. All figures and graphs were designed for readability and accurate data representation. All data underlying the figures are listed in Supplementary Data 2 (for Figs. 1–3 and 5, 6) and 3 (for Fig. 4). Figure 3b was created with SankeyMATIC - github.com/nowthis/sankeymatic.

### Digital technology classification

In line with previous definitions^16^ We define digital phenotyping as the in situ, moment-by-moment quantification of an individual’s phenotype using digital tools, encompassing a broad range of measures from physiological signals like pupil dilation and heart rate, to behavioral traits such as activity and choice bias, and cognitive functions or recent memory recall captured through EMA. We categorized digital technologies into active and passive measurements. Active methods require direct participant involvement, such as responding to prompts, performing cognitive tasks, or self-reporting symptoms. Passive methods automatically collect data to quantify behavioral, physiological, and activity-related measures without requiring active participant involvement. Within these two overarching categories, we defined the following technology-based categories based on commonalities across the included studies:

#### Smartphones

Collect behavioral, cognitive, and mental state data using both active and passive methods. Active measures often rely on EMA, involving user input (e.g., self-reported mood, psychotic symptom ratings). Passive data collection relies heavily on integrated sensors (e.g., GPS-based location tracking, accelerometer data). We also defined the tracking of screen or phone usage as passive, as study-specific data collection is independent of participant engagement.

#### Wearables

Similar to sensor-based smartphone data collection, wearable-based monitoring (e.g., smartwatches, actigraphy wristbands) continuously collects physiological and behavioral data to assess mobility, sleep parameters, and vital signs (e.g., heart rate, electrodermal activity).

#### Social media analysis

Approaches using online platform data to extract linguistic, behavioral, and emotional patterns from online content. Methods include natural language processing (NLP) of posts on platforms such as Twitter, Reddit, Facebook, and Instagram, analyzing features such as word frequency or post content.

#### Audio and video recording

Measurement of vocal and facial expressions, providing information about speech patterns and vocal parameters (e.g., assessing speech fluency) or emotional states through facial features.

#### Eye tracking

Measures visual attention, eye movement dynamics, and emotion recognition by analyzing features such as fixations, saccades, and pupil size. Because all studies used specialized equipment and did not rely on smartphones, this was defined as a separate category.

#### Computerized tests

Digital tests that require active participant participation, designed to evaluate cognitive and mental states, and collected on a laptop, PC, or tablet. For example, they measure parameters such as memory, processing speed, and emotion recognition.

Although, some technologies are traditionally lab-based and often used for point-in-time rather than continuous daily monitoring, these technologies are increasingly portable and accessible for use in naturalistic settings via mobile phones. Including this methodological diversity broadens our review’s scope, offering a richer understanding of the approaches used to assess individuals with SSD. While this diversity requires careful interpretation when comparing findings, it also highlights the potential for integrating various data streams to create more comprehensive digital phenotypes in the future.

Although a few studies utilized EEG and PSG measures, we excluded them from the quantitative comparison analysis between technologies to maintain methodological transparency and enhance the comparability of our results. Because EEG and PSG were not explicitly included in our search strategy, their inclusion in the quantitative comparison could have introduced bias. Nevertheless, to provide a comprehensive descriptive overview, these studies were retained in our general analyses (Figs. 1–3; Supplementary Fig. 1), as they were identified by our search in the context of digital phenotyping.

### Symptom-related domain classification

Included studies were classified into at least one of six symptom-related domains: cognitive performance, physiological signals, behavior and activity, sleep and circadian rhythm, mental state, and digital usage. *Cognitive Performance:* Included assessment of executive function, emotional processing, and social cognition. *Physiological Signals*: Included measures such as ECG characteristics, heart rate, eye tracking, and oximetry during wakefulness. *Behavior and Activity*: Encompassed physical and motor activity, along with measures tracking the environments in which participants spend their time (e.g., at home or in social settings). *Sleep and Circadian Rhythm*: Included parameters recorded exclusively during sleep, such as movement and heart rate. *Mental State*: Included direct reports about the participant’s emotion and mood, alongside traditional SSD symptoms (e.g., emotional withdrawal, suspiciousness, and hallucination events). *Digital Usage*: Assessed measures ranging from message frequency and social media usage to phone status and battery charge.

### Data extraction for pharmacological medication analysis

For this specific analysis, only primary studies that collected and reported primary data on clinical participants were included. Secondary studies relying on published data from other publications were excluded. All primary studies were checked for medication reporting and whether the authors had converted the administered medication into chlorpromazine equivalent doses^62^. Given the wide and variable use of psychotropic and antipsychotic drugs in SSD, conversion to chlorpromazine equivalents allows for a better comparison of average medication intake between studies.

### Effect size analysis between SSD patients and healthy controls

We first analyzed all studies to determine which included a comparison between individuals with SSD and healthy controls (HC). Studies without an HC comparison group were excluded. Next, the studies reporting raw data (mean and standard deviation) for both the SSD and HC groups were included for effect size calculation. Studies without standard deviations were excluded. To quantify group differences, Hedges’g effect sizes were calculated for each of the digital measurements using the pooled standard deviation from both the HC and SSD groups. Hedges’ g was chosen to account for potential bias in studies with small sample sizes. Correspondingly, 95% confidence intervals were computed for each calculated effect size. Our effect size analyses are summarized in Fig. 4. We first present the aggregated mean effect size for each individual study, grouped by symptom domain (Fig. 4a). These study-level means were then averaged to compare across different symptom domains (Fig. 4b) and digital phenotyping technologies (Fig. 4c). Supplementary Fig. 3 provides additional detail: panel A visualizes all individual measurements with a large effect size (g ≥ 0.8), while panels b and c show the mean effect sizes calculated across all 336 individual measurements within each category.

To quantify the consistency of effect sizes across studies, we performed a heterogeneity analysis using the metafor package in R^63^. We calculated the Q statistic, tau², and the I² statistic based on the calculated Hedges’ g values and their corresponding variances. The restricted maximum likelihood method was used to estimate the inter-study variance (tau²), as it has proven to be more reliable, especially for small sample sizes. Both overall heterogeneity across the main symptom domains and subgroup analyses within selected domains were performed to assess the extent of variability and explore potential sources of heterogeneity.

$$g=\frac{{M}_{2}-{M}_{1}}{S{D}_{{pooled}}}\,\,\,\,\,\,\,\,\,\,\,\,\,\,\,S{D}_{{pooled}}=\sqrt{\frac{\left({n}_{1}-1\right)S{D}_{1}^{2}+\left({n}_{2}-1\right)S{D}_{2}^{2}}{\left({n}_{1}+{n}_{2}-2\right)}}$$

To synthesize the effect sizes across studies, we employed a random-effects meta-analysis model. Here, the pooled Hedges’ g was calculated as a weighted average of the individual study effect sizes. Under the random-effects model, the weight assigned to each study $\left({w}_{i}^{* }\right)$ is the inverse of the sum of its within-study variance $\left({v}_{i}=S{E}_{i}^{2}\right)$ and the estimated between-study variance (τ2), i.e., ${w}_{i}^{* }=\frac{1}{\left({v}_{i}+\tau 2\right)}$.

### Comparison between PANSS Scores and Digital Phenotyping

The Positive and Negative Syndrome Scale (PANSS)^64^ is one of the primary diagnostic scales used to assess SSD symptom severity. Therefore, we compared studies that presented diagnostic outcomes of their assessments against baseline PANSS assessments. Other scales, such as the Brief Negative Symptom Scale (BNSS)^65^ and the Brief Psychiatric Rating Scale (BPRS)^66^, were not separately analyzed because fewer than three studies per scale compared them with digital phenotyping measures, and a direct comparison of these scales alongside PANSS scores is difficult to interpret. Studies were included if they reported significant correlations between digital phenotyping measures and PANSS Total, Positive, Negative, or General Scores. From each study, significant correlation coefficients were extracted and assigned to the appropriate PANSS subscale (see also Supplementary Data 2).

### Comparison of psychotic relapse prediction

We examined and compared studies that used machine-learning models to predict psychotic relapse based on digital phenotyping data. The aim was to extract, standardize, and compare key performance measures of the models. First, all relevant publications were identified and their results systematically analyzed. We extracted reported performance measures, including sensitivity, specificity, positive predictive value, negative predictive value, F2 score, accuracy, precision, recall, Receiver Operating Characteristic Area Under the Curve (ROC AUC), Precision-Recall Area Under the Curve (PR AUC), and mean AUC. For comparability, ROC AUC was approximated using the method of Zhang and Mueller (2005)^67^ when only sensitivity and specificity were available; however, we acknowledge this estimation introduces uncertainty and may not reflect the true AUC value. It is methodologically important to note that for datasets with scarce events, such as psychotic relapse (Supplementary Table 2), performance on class imbalance is critical^68^. Finally, we also categorized the types of input data used for relapse prediction in each study based on the symptom domain classification.

### Risk of bias assessment

In all studies, we evaluated reporting and methodological limitations. Due to the heterogeneity across studies in study designs and methodological approaches, a formalized risk of bias assessment tool could not be applied. We assessed overall quality based on six criteria (adapted from AXIS and JBI) relevant across the studies^69,70^: (1) clarity of eligibility criteria (e.g., inclusion/exclusion criteria); (2) potential reporting bias in the results section (e.g., selective reporting); (3) missing data handling description; (4) description of recruitment procedure; (5) justification of sample size and (6) adherence reporting.

Each study was rated on a 3-point scale for each criterion: 0 = not reported, 1 = partially reported, and 2 = fully reported; each assessment was done independently by at least 2 co-authors, assessment disagreements were resolved by all co-authors together. An additional risk of bias assessment for the relapse prediction studies was performed based on 25 items of the PROBAST + AI guidelines^37^. A composite sum score based on the same scoring as above was calculated based on the following PROBAST + AI items: 5a,b, 7, 8a-b, 9a-b, 10, 11, 12a-g, 13, 14, 15, 16, 20b-c, 21, 22, 23a. English- and German-language studies were included, which may have resulted in the exclusion of relevant studies published in other languages. As only peer-reviewed studies were included, preprints and other relevant gray literature were not considered, potentially excluding newer research.

## Supplementary information

Supplementary Information

Supplementary Data

## Data Availability

All data generated or analyzed during this study are included in this published article and its supplementary information files as well as the supporting data files available at 10.17605/OSF.IO/86VNG.
