# Supplementary material for: Diagnostic digital phenotyping in schizophrenia-spectrum disorders: a systematic review
Source: NPJ Digit Med. 2025 Dec 1;9:16. doi: 10.1038/s41746-025-02194-w (PMC12779945; doi:10.1038/s41746-025-02194-w)
Supplement: Supplementary file 1 — Supplementary Information [file 41746_2025_2194_MOESM1_ESM.pdf]

Supplementary Information

This supplementary file provides additional materials that support the findings of the main manuscript. It includes the 27-item PRISMA checklist, outlining adherence to systematic review reporting standards; a visual representation of the study selection process (Supplementary Figure 1); an overview of the risk of bias assessment for all studies (Supplementary Figure 2a) and a specialized assessment on relapse prediction studies (Supplementary Figure 2b); Supplementary Figure 3 reports on effect size comparison based on individual diagnostic measurements; the results of the heterogeneity analysis across symptom domains (Supplementary Table 1); a detailed list of the search terms used in the databases to identify the included literature.

PRISMA 2020 Checklist

Section and Topic	Item #	Checklist item	Location where item is reported
**TITLE**			
Title	1	Identify the report as a systematic review.	Page 1
**ABSTRACT**			
Abstract	2	See the PRISMA 2020 for Abstracts checklist.	Page 2
**INTRODUCTION**			
Rationale	3	Describe the rationale for the review in the context of existing knowledge.	Pages 3-4
Objectives	4	Provide an explicit statement of the objective(s) or question(s) the review addresses.	Final paragraph of introduction, Page 4
**METHODS**			
Eligibility criteria	5	Specify the inclusion and exclusion criteria for the review and how studies were grouped for the syntheses.	Page 5
Information sources	6	Specify all databases, registers, websites, organisations, reference lists and other sources searched or consulted to identify studies. Specify the date when each source was last searched or consulted.	Pages 4-5
Search strategy	7	Present the full search strategies for all databases, registers and websites, including any filters and limits used.	Pages 4 -5, detailed search terms for each database in supplementary material
Selection process	8	Specify the methods used to decide whether a study met the inclusion criteria of the review, including how many reviewers screened each record and each report retrieved, whether they worked independently, and if applicable, details of automation tools used in the process.	Page 5
Data collection process	9	Specify the methods used to collect data from reports, including how many reviewers collected data from each report, whether they worked independently, any processes for obtaining or confirming data from study investigators, and if applicable, details of automation tools used in the process.	Pages 5-6
Data items	10a	List and define all outcomes for which data were sought. Specify whether all results that were compatible with each outcome domain in each study were sought (e.g. for all measures, time points, analyses), and if not, the methods used to decide which results to collect.	Pages 6-8
	10b	List and define all other variables for which data were sought (e.g. participant and intervention characteristics, funding sources). Describe any assumptions made about any missing or unclear information.	Pages 5-6

PRISMA 2020 Checklist

Section and Topic	Item #	Checklist item	Location where item is reported
Study risk of bias assessment	11	Specify the methods used to assess risk of bias in the included studies, including details of the tool(s) used, how many reviewers assessed each study and whether they worked independently, and if applicable, details of automation tools used in the process.	Page 11
Effect measures	12	Specify for each outcome the effect measure(s) (e.g. risk ratio, mean difference) used in the synthesis or presentation of results.	Pages 9-11
Synthesis methods	13a	Describe the processes used to decide which studies were eligible for each synthesis (e.g. tabulating the study intervention characteristics and comparing against the planned groups for each synthesis (item #5)).	Pages 5-8
	13b	Describe any methods required to prepare the data for presentation or synthesis, such as handling of missing summary statistics, or data conversions.	Pages 9-11
	13c	Describe any methods used to tabulate or visually display results of individual studies and syntheses.	Pages 5-6
	13d	Describe any methods used to synthesize results and provide a rationale for the choice(s). If meta-analysis was performed, describe the model(s), method(s) to identify the presence and extent of statistical heterogeneity, and software package(s) used.	Pages6-10
	13e	Describe any methods used to explore possible causes of heterogeneity among study results (e.g. subgroup analysis, meta-regression).	Pages 9-10
	13f	Describe any sensitivity analyses conducted to assess robustness of the synthesized results.	No sensitivity analysis, not applicable
Reporting bias assessment	14	Describe any methods used to assess risk of bias due to missing results in a synthesis (arising from reporting biases).	Page 11
Certainty assessment	15	Describe any methods used to assess certainty (or confidence) in the body of evidence for an outcome.	Confidence intervals were reported for all effect size estimates to indicate the precision of findings, Page 9
**RESULTS**			
Study selection	16a	Describe the results of the search and selection process, from the number of records identified in the search to the number of studies included in the review, ideally using a flow diagram.	Pages 11-12 and flow diagram in supplementary figure 1
	16b	Cite studies that might appear to meet the inclusion criteria, but which were excluded, and explain why they were excluded.	Supplementary figure 1 – no study cited, see figure legend
Study characteristics	17	Cite each included study and present its characteristics.	Supplementary Data 1
Risk of bias in studies	18	Present assessments of risk of bias for each included study.	Supplementary Data 1 risk of bias scores and summary figures in Suppl. Figure 2a, and 2b, ant Suppl. Table 2

PRISMA 2020 Checklist

Section and Topic	Item #	Checklist item	Location where item is reported
Results of individual studies	19	For all outcomes, present, for each study: (a) summary statistics for each group (where appropriate) and (b) an effect estimate and its precision (e.g. confidence/credible interval), ideally using structured tables or plots.	Results Section 11-24 & Supplementary Data 2,3 and supplementary table 1
Results of syntheses	20a	For each synthesis, briefly summarise the characteristics and risk of bias among contributing studies.	Overall Risk of bias assessment Pages 23 and 24, discussion Page 28; Supplementary figure 2a and 2b
	20b	Present results of all statistical syntheses conducted. If meta-analysis was done, present for each the summary estimate and its precision (e.g. confidence/credible interval) and measures of statistical heterogeneity. If comparing groups, describe the direction of the effect.	Results section, Pages 16- 23
	20c	Present results of all investigations of possible causes of heterogeneity among study results.	Page 17, presentation of heterogeneity
	20d	Present results of all sensitivity analyses conducted to assess the robustness of the synthesized results.	No sensitivity analysis conducted
Reporting biases	21	Present assessments of risk of bias due to missing results (arising from reporting biases) for each synthesis assessed.	Supplementary Data 1; supplementary figure 2a)
Certainty of evidence	22	Present assessments of certainty (or confidence) in the body of evidence for each outcome assessed.	Figure 4, Supplementary Figure 3, Supplementary Data 3
**DISCUSSION**			
Discussion	23a	Provide a general interpretation of the results in the context of other evidence.	Pages 24-25
	23b	Discuss any limitations of the evidence included in the review.	Throughout the discussion:, Page 24-28
	23c	Discuss any limitations of the review processes used.	Discussion Pages 26-28
	23d	Discuss implications of the results for practice, policy, and future research.	Conclusion - Pages 28 and 29
**OTHER INFORMATION**			
Registration and protocol	24a	Provide registration information for the review, including register name and registration number, or state that the review was not registered.	NA
	24b	Indicate where the review protocol can be accessed, or state that a protocol was not prepared.	NA
	24c	Describe and explain any amendments to information provided at registration or in the protocol.	NA
Support	25	Describe sources of financial or non-financial support for the review, and the role of the funders or sponsors in the review.	Acknowledgements
Competing interests	26	Declare any competing interests of review authors.	Disclosure statement

Availability of data, code and other materials	27	Report which of the following are publicly available and where they can be found: template data collection forms; data extracted from included studies; data used for all analyses; analytic code; any other materials used in the review.	All data analyzed are included in this article and its supplementary material

From: Page MJ, McKenzie JE, Bossuyt PM, Boutron I, Hoffmann TC, Mulrow CD, et al. The PRISMA 2020 statement: an updated guideline for reporting systematic reviews. BMJ 2021;372:n71. doi: 10.1136/bmj.n71. This work is licensed under CC BY 4.0. To view a copy of this license, visit https://creativecommons.org/licenses/by/4.0/

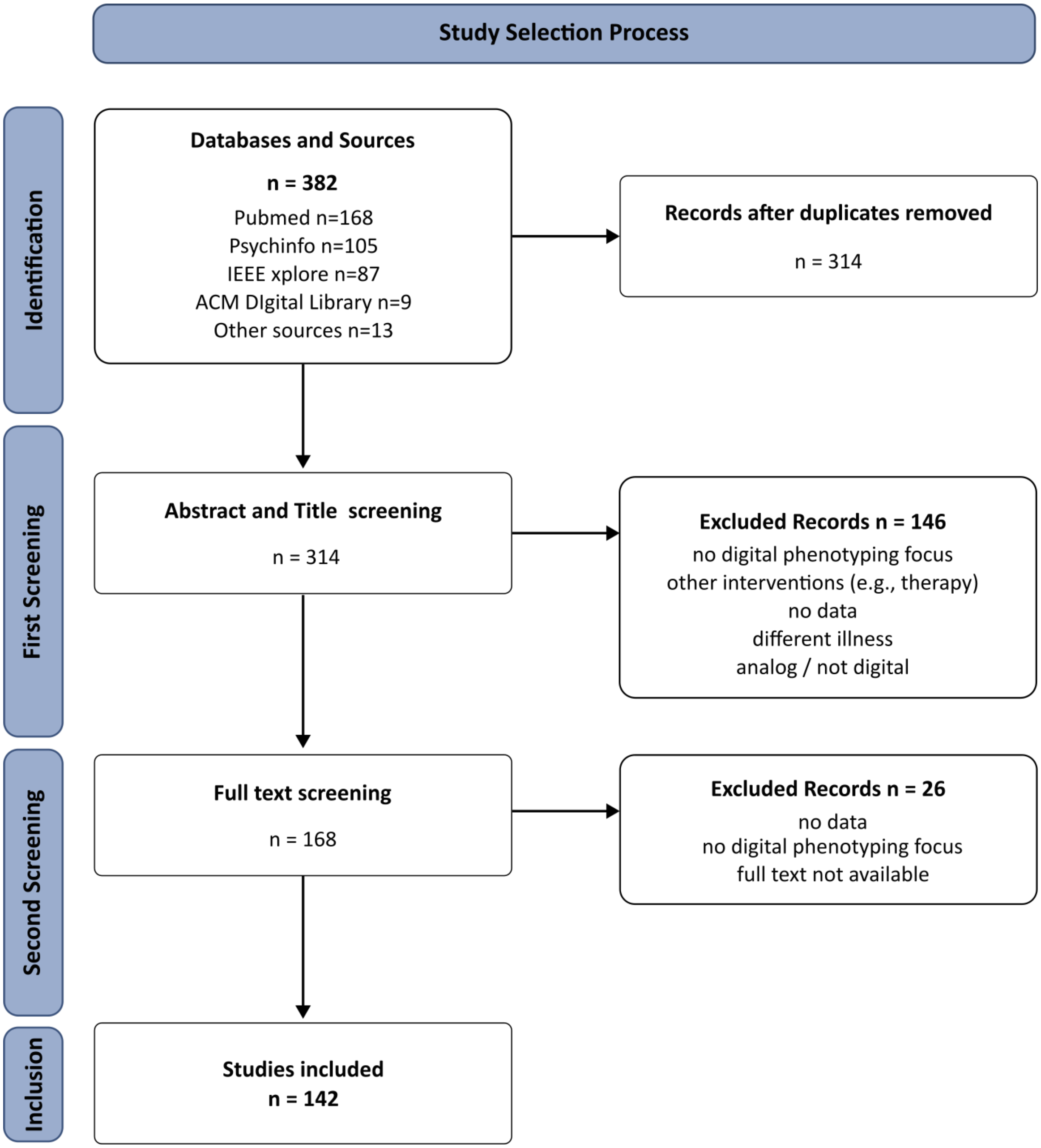

Supplementary Figure 1: Selection process for systematic review. The flow diagram based on PRISMA shows the selection of the studies. A total of 142 studies met all inclusion criteria and were included in the analysis. Note: the exclusion categories are listed in descending order of frequency. This because many studies were excluded for multiple reasons (e.g. a study may not use digital methods and does not show any data at the same time). Therefore, no precise breakdown by category is provided to avoid misinterpretation.

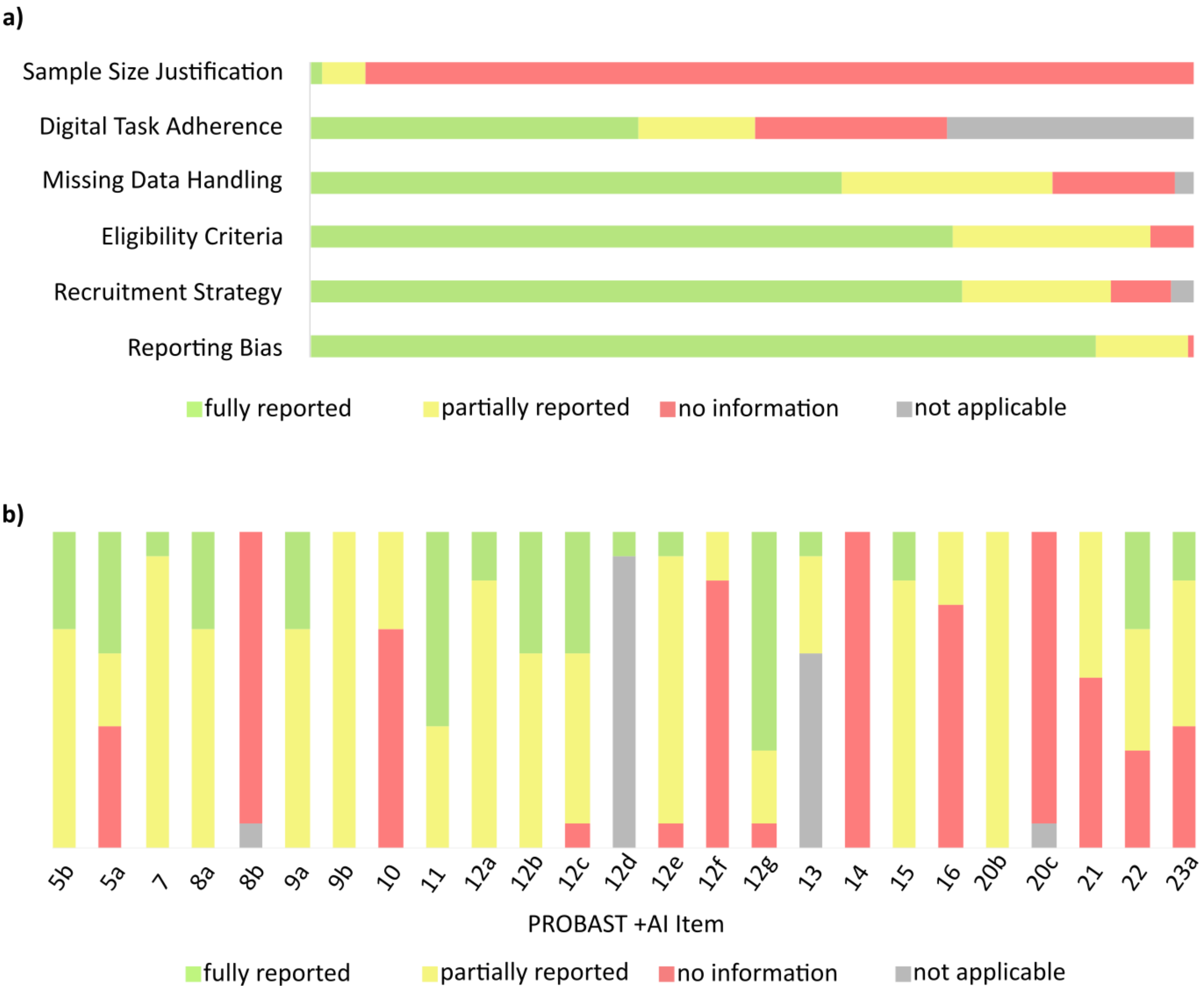

Supplementary Figure 2 – Methodological quality and risk of bias assessment. (a) Assessment of reporting quality for all included studies (n=142). The horizontal bar chart summarizes reporting completeness across six key methodological criteria: sample size justification, adherence, missing data handling, eligibility criteria, recruitment strategy, and reporting bias. (b) Risk of bias assessment for relapse prediction studies (n=13). The vertical bar chart summarizes reporting based on 25 selected items from the PROBAST+AI guidelines. In both charts, items were rated as fully reported (green), partially reported (yellow), no information provided (red), or not applicable (gray).

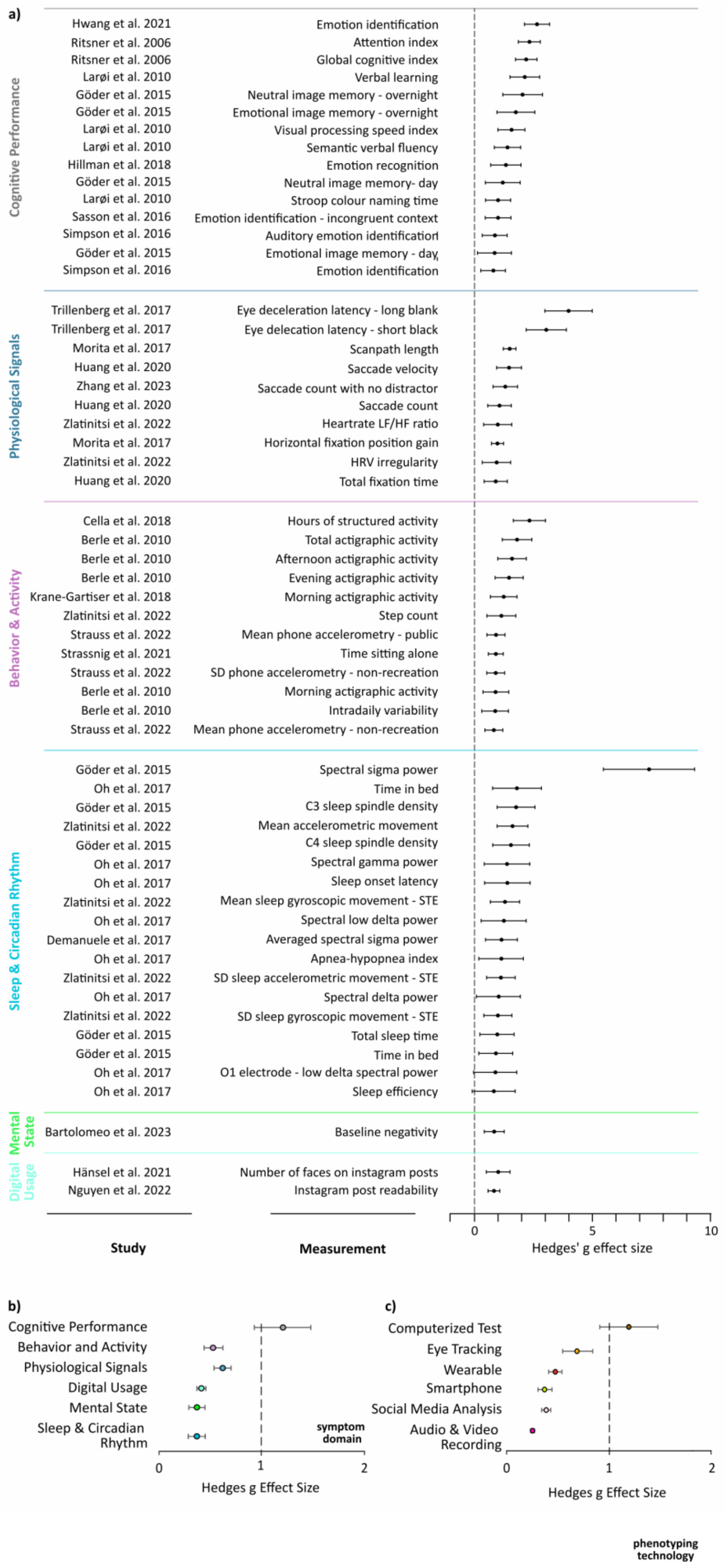

Supplementary Figure 3 - Effect size comparison based on individual diagnostic measurements. (a) Individual diagnostic measures with a g ≥ 0.8 ordered by effect size and grouped into six domains: Cognitive Performance (n = 35, dark purple), Physiological Signals (n = 77, dark blue), Behavior & Activity (n = 68, light purple), Sleep & Circadian Rhythm (n = 55, light blue), Mental State (n = 24, green), and Digital Usage (n = 77, light cyan). All remaining measurements with effect size below 0.8 are found in Supplementary Data 3. (b) Weighted average effect size for each symptom-related domain based on individual measurements, not study effects. (c) Weighted average effect size for each phenotyping technology based on individual measurements, not study effects. Note: data as mean + CI; all effect sizes are shown as an absolute (positive) value for visual clarity and ease of comparison, regardless of the original effect direction. LF = low frequency, HF = high frequency, HRV = heart rate variability, SD = standard deviation, STE = short time energy.

Supplementary Table 1: Heterogeneity statistics by symptom domain and subdomain. Overall heterogeneity for each main domain is shown in **black**; detailed subgroup analyses for corresponding subdomains are shown in gray.

symptom-domain	Q	I^2	df	tau	tau^2	p-value
**Behavior and Activity**	**208.8502**	**70.1%**	**67**	**0.326031**	**0.106296**	**<0,001**
physical activity and movement	99.0387	82.0%	22	0.453206	0.205396	<0,001
**Cognitive Performance**	**732.7176**	**93.0%**	**34**	**0.637345**	**0.406208**	**<0,001**
attention and reaction	93.2855	91.5%	8	0.746377	0.557079	<0,001
emotion processing	191.7618	96.1%	9	0.806120	0.649380	<0,001
**Mental state**	**39.6470**	**51.0%**	**23**	**0.120484**	**0.014516**	**0.017**
negative experience	11.6169	40.0%	7	0.177150	0.031382	0.114
**Physiological Signals**	**470.9736**	**89.5%**	**76**	**0.413615**	**0.170776**	**<0,001**
eye tracking measures	406.9200	94.0%	41	0.575420	0.331108	<0,001
**Sleep and Circadian Rhythm**	**96.3577**	**41.1%**	**54**	**0.214512**	**0.046015**	**<0,001**
sleep architecture	10.1354	5.4%	9	0.055418	0.003071	0.340
**Digital Usage**	**254.1913**	**32.7%**	**76**	**0.238836**	**0.057042**	**<0,001**

Supplementary Table 2: Risk of Bias Relapse Prediction Overview. The table presents the number of included patients, the reported number of outcome events (relapse events) as well as the summary score PROBAST +AI per study. (Note: Relapse events with ">" indicate minimal values as exact numbers could not be inferred from the primary data.)

Study	Nr. of individuals with SSD	Nr. of outcomes (relapse events)	PROBAST+AI Summary Score
Henson et al. 2021	63	29	19
Górzyn′ski et al 2024	9	>9	15
Adler et al. 2020	60	18	31
Kaliosis et al.2024	9	>9	20
Cohen et al. 2023	76	23	25
Avramidis et al.2024	10	>10	17
Zlatintsi et al.2022	10	>10	23
Hein et al.2024	9	>9	18
Birnbaum et al.2019	51	124	30
Zlatintsi et al.2023	10	>10	18
Wu et al.2024	9	>9	16
Mallol-Ragolta et al. 2024	9	>9	15
Fekas et al. 2023	38	37	23

Supplementary Information 1: Search terms in databases on November 28, 2024:

PUBMED (n = 168)

("Schizophrenia"[Mesh] OR "Schizophrenia Spectrum and Other Psychotic Disorders"[Mesh] OR "schizophren*"[Title] OR "psychosis*"[Title] OR "psychotic disorder"[Title] OR "schizotyp*"[Title] OR"psychotic*"[Title] OR "schizophren*"[Other Term] OR "psychosis*"[Other Term] OR "psychotic disorder"[Other Term] OR "schizotyp*"[Other Term] OR "psychotic*"[Other Term]) **AND** ("mhealth"[Title] OR "smartphone*"[Title] OR "smartwatch*"[Title] OR "wearable*"[Title] OR "actigraphy"[Title] OR "digital phenotyp*"[Title] OR "digital-phenotyp*"[Title] OR "sleep"[Title] OR "social media"[Title] OR "screen time"[Title] OR "eye-tracking"[Title] OR "eye tracking"[Title] OR "Digital Health"[Mesh] OR "Ecological Momentary Assessment"[Mesh] OR "Smartphone"[Mesh] OR "Mobile Applications"[Mesh] OR "Mobile Applications"[Title] OR "Mobile Applications"[Other Term] OR "cell phone"[Title] OR "ecological momentary assessment"[Title] OR "mhealth"[Other Term] OR "smartphone*"[Other Term] OR "smartwatch*"[Other Term] OR "wearable*"[Other Term] OR "actigraphy"[Other Term] OR "sleep"[Other Term] OR "social media"[Other Term] OR "screen time"[Other Term] OR "eye-tracking"[Other Term] OR "eye tracking"[Other Term] OR "cell phone"[Other Term] OR "ecological momentary assessment"[Other Term] OR "computerized"[Title] OR "computerized"[Other Term]) **AND** ("digital phenotyp*"[Title] OR "digital-phenotyp*"[Title] OR "diagnosis"[Title] OR "diagnos*"[Title] OR "predict*"[Title] OR "identif*"[Title] OR "recogn*"[Title] OR "detect*"[Title] OR "digital phenotyp*"[Other Term] OR "digital-phenotyp*"[Other Term] OR "diagnosis"[Other Term] OR "diagnos*"[Other Term] OR "predict*"[Other Term] OR "character*"[Title] OR "character*"[Other Term] OR "identif*"[Other Term] OR "recogn*"[Other Term] OR "symptom monitoring"[Other Term] OR "symptom monitoring"[Title] OR "detect*"[Other Term]) NOT review

PSYCHINFO (n = 105)

DE "Acute Schizophrenia" OR DE "Catatonic Schizophrenia" OR DE "Childhood Onset Schizophrenia" OR DE "Process Schizophrenia" OR DE "Schizoaffective Disorder" OR DE "Undifferentiated Schizophrenia" OR DE "Positive and Negative Symptoms" OR DE "Schizoid Personality Disorder" OR DE "Schizophrenia (Disorganized Type)" OR DE "Schizophreniform Disorder" OR DE "Schizotypal Personality Disorder" OR DE "Schizotypy" OR DE "Psychosis" OR DE "Affective Psychosis" OR DE "Alcohol Induced Psychotic Disorders" OR DE "Brief Psychotic Disorder" OR DE "Childhood Onset Psychosis" OR DE "Chronic Psychosis" OR DE "Paranoid Psychosis" OR DE "Postpartum Psychosis" OR DE "Reactive Psychosis" OR DE "Schizophrenia" OR DE "Substance Induced Psychotic Disorders" OR DE "Paranoid Schizophrenia" OR "schizophren*" OR "psychosis*" OR "psychotic disorder" OR "schizotyp*" OR "psychotic*" OR "psychotic episode" **AND** DE "Smartphones" OR DE "Physical Mobility" OR DE "Sensor Technology" OR DE "Sleep" OR DE "Actigraphy" OR DE "Physical Activity" OR DE "Sleep Wake Cycle" OR DE "Social Media" OR DE "Computer Usage" OR DE "Smartphone Use" OR DE "Digital Gaming" OR DE "Gamification" OR DE "Screen Time" OR DE "Facial Recognition (Artificial Intelligence)" OR DE "Ecological Momentary Assessment" OR DE "Mobile Phones" OR DE "Mobile Health" OR DE "Mobile Applications" OR DE "Mobile Devices" OR DE "Mobile Technology" OR DE "Wearable Devices" OR "mhealth" OR "smartphone*" OR "smartwatch*" OR "wearable*" OR "actigraphy" OR "sleep" OR "social media" OR "screen time" OR "eye-tracking" OR "eye tracking" OR "digital phenotyp*" OR "digital-phenotyp*" OR "cell phone" OR "ecological momentary assessment" **AND** DE "Diagnosis" OR DE "Prediction" OR DE "Prognosis" OR DE "Classification (Machine Learning)" OR "digital phenotyp*" OR "digital-phenotyp*" OR "diagnosis" OR "diagnos*" OR "predict*" OR "identif*" OR "recogni*" OR "detect*" NOT review

ACM DIGITAL LIBRARY (n = 9)

("Acute Schizophrenia" OR "Catatonic Schizophrenia" OR "Childhood Onset Schizophrenia" OR "Process Schizophrenia" OR "Schizoaffective Disorder" OR "Undifferentiated Schizophrenia" OR "Positive and Negative Symptoms" OR "Schizoid Personality Disorder" OR "Schizophrenia (Disorganized Type)" OR "Schizophreniform Disorder" OR "Schizotypal Personality Disorder" OR "Schizotypy" OR "Psychosis" OR "Affective Psychosis" OR "Alcohol Induced Psychotic Disorders" OR "Brief Psychotic Disorder" OR "Childhood Onset Psychosis" OR "Chronic Psychosis" OR "Paranoid Psychosis" OR "Postpartum Psychosis" OR "Reactive Psychosis" OR "Schizophrenia" OR "Substance Induced Psychotic Disorders" OR "Paranoid Schizophrenia" OR "schizophren*" OR "psychosis*" OR "psychotic disorder" OR "schizotyp*" OR "psychotic*" OR "psychotic episode") **AND** ("Smartphones" OR "Physical Mobility" OR "Sensor Technology" OR "Sleep" OR "Actigraphy" OR "Physical Activity" OR "Sleep Wake Cycle" OR "Social Media" OR "Computer Usage" OR "Smartphone Use" OR "Digital Gaming" OR "Gamification" OR "Screen Time" OR "Facial Recognition (Artificial Intelligence)" OR "Ecological Momentary Assessment" OR "Mobile Phones" OR "Mobile Health" OR "Mobile Applications" OR "Mobile Devices" OR "Mobile Technology" OR "Wearable Devices" OR "mhealth" OR "smartphone*" OR "smartwatch*" OR "wearable*" OR "actigraphy" OR "sleep" OR "social media" OR "screen time" OR "eye-tracking" OR "eye tracking" OR "digital phenotyp*" OR "digital-phenotyp*" OR "cell phone" OR "ecological momentary assessment") **AND** ("Diagnosis" OR "Prediction" OR "Prognosis" OR "Classification (Machine Learning)" OR "digital phenotyp*" OR "digital-phenotyp*" OR "diagnosis" OR "diagnos*" OR "predict*" OR "identif*" OR "recogni*" OR "detect*")

IEEE XPLORE (n = 87)

("Index Terms":schizophren* OR "Index Terms":schizophrenia OR "Index Terms":schizotyp* OR "Index Terms":psychosis OR "Index Terms":psychotic disorder OR "Index Terms":psychotic) AND ("Index Terms":mhealth OR "Index Terms":smartphone OR "Index Terms":smartphones OR "Index Terms":smartwatch OR "Index Terms":smartwatches OR "Index Terms":wearable OR "Index Terms":wearables OR "Index Terms":actigraphy OR "Index Terms":digital phenotype OR "Index Terms":digital phenotyping OR "Index Terms":sleep OR "Index Terms":social media OR "Index Terms":screen time OR "Index Terms":eye-track* OR "Index Terms":eye track* OR "Index Terms":ecological momentary assessment OR "Index Terms":smartphone OR "Index Terms":mobile application*) AND ("Index Terms":digital phenotype OR "Index Terms":digital phenotyping OR "Index Terms":diagnos* OR "Index Terms":prediction OR "Index Terms":predict OR "Index Terms":identify OR "Index Terms":identification OR "Index Terms":recogn* OR "Index Terms":detect* OR "Index Terms":character* OR "Index Terms":symptom monitoring NOT "Index Terms":review)
